# Glucose Biosensors: An Overview of Use in Clinical Practice

**DOI:** 10.3390/s100504558

**Published:** 2010-05-04

**Authors:** Eun-Hyung Yoo, Soo-Youn Lee

**Affiliations:** 1 Department of Laboratory Medicine, Konyang University Hospital, College of Medical Science, Konyang University, Daejon, Korea; E-Mail: yooeh@kyuh.co.kr; 2 Department of Laboratory Medicine and Genetics, Samsung Medical Center, Sungkyunkwan University School of Medicine, Seoul, Korea

**Keywords:** diabetes mellitus, glucose biosensor, point-of-care testing, performance, self-monitoring of blood glucose

## Abstract

Blood glucose monitoring has been established as a valuable tool in the management of diabetes. Since maintaining normal blood glucose levels is recommended, a series of suitable glucose biosensors have been developed. During the last 50 years, glucose biosensor technology including point-of-care devices, continuous glucose monitoring systems and noninvasive glucose monitoring systems has been significantly improved. However, there continues to be several challenges related to the achievement of accurate and reliable glucose monitoring. Further technical improvements in glucose biosensors, standardization of the analytical goals for their performance, and continuously assessing and training lay users are required. This article reviews the brief history, basic principles, analytical performance, and the present status of glucose biosensors in the clinical practice.

## 1. Introduction

Diabetes mellitus is the most common endocrine disorder of carbohydrate metabolism. Worldwide, it is a leading cause of morbidity and mortality and a major health problem for most developed societies. The prevalence of diabetes continues to increase. The crude estimated prevalence of diabetes in adults in the United States (US) has been reported to be 9.6% (20.4 million) in 2003–2006 [1]. Moreover, it is predicted that 48.3 million people in the US will have diabetes by 2050 [2]. The World Health Organization (WHO) has put the number of persons with diabetes worldwide at approximately 171 million in 2000, and this is expected to increase to 366 million by 2030 [3]. A recent study estimated that the world prevalence of diabetes among adults (20–79 years of age) would be 6.4%, affecting 285 million adults in 2010 [4]. And it will increase to 7.7%, affecting 439 million adults by 2030. A sedentary lifestyle combined with changes in eating habits and the increasing frequency of obesity is thought to be the major causes of such increased rates.

Multiple laboratory tests are used for the diagnosis and management of patients with diabetes. The blood glucose concentration is the major diagnostic criterion for diabetes with HbA1c level [5] and is a useful tool for patient monitoring. Self-monitoring of blood glucose (SMBG) has been established as a valuable tool for the management of diabetes [6–12]. The goal of SMBG is to help the patient achieve and maintain normal blood glucose concentrations in order to delay or even prevent the progression of microvascular (retinopathy, nephropathy and neuropathy) and macrovascular complications (stroke and coronary artery disease). The findings of the Diabetes Control and Complications Trial (DCCT) and the United Kingdom Prospective Diabetes Study (UKPDS) clearly showed that intensive control of elevated levels of blood glucose in patients with diabetes, decreases the frequency of complications such as nephropathy, neuropathy, and retinopathy, and may reduce the occurrence and severity of large blood vessel disease [13–15]. In addition, it can also be useful for detecting hypoglycemia and providing real-time information for adjusting medications, dietary regimens, and physical activity in order to achieve glycemic goals [10,16]. Regular and frequent measurement of blood glucose may provide data for optimizing and/or changing patient treatment strategies.

According to the recommendations of the ADA, SMBG should be used in patients on intensive insulin therapy (at least three times daily). And it may useful in patients using less frequent insulin injections, noninsulin therapies, or medical nutrition therapy alone [16].

Due to such recommendations for maintaining normal blood glucose levels, a series of suitable glucose-measuring devices have been developed. Biosensor technology has developed rapidly and can play a key role providing a powerful analytical tool with major applications particularly in medicine. Today’s biosensor market is dominated by glucose biosensors. In 2004, glucose biosensors accounted for approximately 85% of the world market for biosensors, which had been estimated to be around $5 billion USD [17]. The glucose biosensor market growth is accelerating and manufacturers are engaged in fierce competition. According to the recent report by Global Industry Analysts, Inc., the global market for glucose biosensors and strips will reach $11.5 billion USD by 2012.

This article reviews the brief history of biosensors, basic principles of operation, analytical performance requirements, and the present status of glucose biosensors. In addition, how to assess the reliability of testing in clinical practice will be discussed.

## 2. Basic Principles of Glucose Biosensors

A biosensor can be defined as a “compact analytical device or unit incorporating a biological or biologically derived sensitive recognition element integrated or associated with a physio-chemical transducer” [18]. There are three main parts of a biosensor: (i) the biological recognition elements that differentiate the target molecules in the presence of various chemicals, (ii) a transducer that converts the biorecognition event into a measurable signal, and (iii) a signal processing system that converts the signal into a readable form [19–21]. The molecular recognition elements include receptors, enzymes, antibodies, nucleic acids, microorganisms and lectins [22,23]. The five principal transducer classes are electrochemical, optical, thermometric, piezoelectric, and magnetic [24]. The majority of the current glucose biosensors are of the electrochemical type, because of their better sensitivity, reproducibility, and easy maintenance as well as their low cost. Electrochemical sensors may be subdivided into potentiometric, amperometric, or conductometric types [25–27]. Enzymatic amperometric glucose biosensors are the most common devices commercially available, and have been widely studied over the last few decades. Amperometric sensors monitor currents generated when electrons are exchanged either directly or indirectly between a biological system and an electrode [28,29].

Generally, glucose measurements are based on interactions with one of three enzymes: hexokinase, glucose oxidase (GOx) or glucose-1-dehydrogenase (GDH) [30,31]. The hexokinase assay is the reference method for measuring glucose using spectrophotometry in many clinical laboratories [32]. Glucose biosensors for SMBG are usually based on the two enzyme families, GOx and GDH. These enzymes differ in redox potentials, cofactors, turnover rate and selectivity for glucose [33]. GOx is the standard enzyme for biosensors; it has a relatively higher selectivity for glucose. GOx is easy to obtain, cheap, and can withstand greater extremes of pH, ionic strength, and temperature than many other enzymes, thus allowing less stringent conditions during the manufacturing process and relatively relaxed storage norms for use by lay biosensor users [33,34].

The basic concept of the glucose biosensor is based on the fact that the immobilized GOx catalyzes the oxidation of β-D-glucose by molecular oxygen producing gluconic acid and hydrogen peroxide [35]. In order to work as a catalyst, GOx requires a redox cofactor—flavin adenine dinucleotide (FAD). FAD works as the initial electron acceptor and is reduced to FADH2.

$$\text{Glucose}+\text{GOx}-{\text{FAD}}^{+}\to \text{Glucolactone}+\text{GOx}-{\text{FADH}}_{2}$$

The cofactor is regenerated by reacting with oxygen, leading to the formation of hydrogen peroxides.

$$\text{GOx}-{\text{FADH}}_{2}+O_{2}\to \text{GOx}-\text{FAD}+H_{2}\;O_{2}$$

Hydrogen peroxide is oxidized at a catalytic, classically platinum (Pt) anode. The electrode easily recognizes the number of electron transfers, and this electron flow is proportional to the number of glucose molecules present in blood [36].

$$H_{2}O_{2}\to {2H}^{+}+O_{2}+2e$$

Three general strategies are used for the electrochemical sensing of glucose; by measuring oxygen consumption, by measuring the amount of hydrogen peroxide produced by the enzyme reaction or by using a diffusible or immobilized mediator to transfer the electrons from the GOx to the electrode. The number and types of GDH-based amperometric biosensors have been increasing recently. The GDH family includes GDH-pyrroquinolinequinone (PQQ) [37–39] and GDH-nicotinamide-adenine dinucleotide (NAD) [40–42]. The enzymatic reaction of GDH is independent of the dissolved oxygen. The quinoprotein GDH recognition element uses PQQ as a cofactor.

Glucose+PQQ(ox)→gluconolactone+PQQ(red)

This mechanism requires neither oxygen nor NAD^+^. GDH-PQQ is a particularly efficient enzyme system, with a rapid electron transfer rate, but it is relatively expensive [17].

GDH with NAD as a cofactor produces NADH rather than H_2_O_2_. NAD is a major electron acceptor in the oxidation of glucose, during which the nicotinamide ring of NAD^+^ accepts a hydrogen ion and two electrons, equivalent to a hydride ion. The reduced form of this carrier generated in this reaction is called NADH, which can be electrochemically oxidized.

$$\begin{array}{c} \text{Glucose}+{\text{NAD}}^{+}\to \text{gluconolactone}+\text{NADH}\\ \mathrm{NADH}\to {\mathrm{NAD}}^{+}+H^{+}+2e \end{array}$$

## 3. Historical Perspectives of Glucose Biosensors

Although a variety of glucose sensors are available, the glucose biosensor has changed little in principle over several years (Table 1). However, the first blood glucose meter was not a biosensor. It was the Ames Reflectance Meter (ARM) (Miles Laboratories, Elkhart, IN, USA) based on a reflectometer and the Dextrostix introduced in 1971. Dextrostix, the first blood glucose strip, had been available since 1965, and was originally designed to show color changes [43–46]; the blood sample was gently washed off after one minute, before inserting the strip into the meter. Although the ARM was expensive and cumbersome to use, it replaced the Ames Eyetone glucose analyzer. Early versions of glucose-sensing devices were based on the reflectometer.

### 3.1. First-generation of Glucose Biosensors

The concept of the biosensor for measuring glucose levels was first proposed in 1962 by Clark and Lyons from the Children’s Hospital of Cincinnati [19]. This glucose biosensor was composed of an oxygen electrode, an inner oxygen semipermeable membrane, a thin layer of GOx, and an outer dialysis membrane. Enzymes could be immobilized at an electrochemical detector to form an enzyme electrode. A decrease in the measured oxygen concentration was proportional to the glucose concentration. Updike and Hicks significantly simplified the electrochemical glucose assay by immobilizing and thereby stabilizing GOx [20,47]. They immobilized GOx in a polyacrylamide gel on an oxygen electrode for the first time and measured glucose concentration in biological fluids [20].

The first commercially successful glucose biosensor using Clark’s technology was the Yellow Springs Instrument Company analyzer (Model 23A YSI analyzer) for the direct measurement of glucose in 1975, which was based on the amperometric detection of hydrogen peroxide. This analyzer was almost exclusively used in clinical laboratories because of its high cost due to the expensive platinum electrode.

The first-generation glucose biosensors were based on the use of natural oxygen substrate and on the detection of the hydrogen peroxide produced. Measurements of peroxide formation have the advantage of being simpler, especially when miniature devices are being considered [48]. However, the main problem with the first-generation of glucose biosensors was that the amperometric measurement of hydrogen peroxide required a high operation potential for high selectivity.

Considerable efforts during the late 1980s were devoted to minimize the interference of endogenous electroactive species, such as ascorbic acid, uric acid, and certain drugs. Another drawback was the restricted solubility of oxygen in biological fluids, which produced fluctuations in the oxygen tension, known as the “oxygen deficit” [49].

### 3.2. Second-generation of Glucose Biosensors

The abovementioned limitations of the first-generation glucose biosensors were overcome by using mediated glucose biosensors, *i.e.*, second-generation glucose sensors. The improvements were achieved by replacing oxygen with non-physiological electron acceptors, called redox mediators that were able to carry electrons from the enzyme to the surface of the working electrode [49]. A reduced mediator is formed instead of hydrogen peroxide and then reoxidized at the electrode, providing an amperometric signal and regenerating the oxidized form of the mediator [28]. A variety of electron mediators, such as ferrocene, ferricyanide, quinines, tetrathialfulvalene (TTF), tetracyanoquinodimethane (TCNQ), thionine, methylene blue, and methyl viologen were used to improve sensor performance [50–53]. Ferrocenes fit all criteria for a good mediator, such as not reacting with oxygen, stable in both the oxidized and reduced forms, independent of pH, showing reversible electron transfer kinetics, and reacting rapidly with the enzyme [53]. They were extensively studied as electron-shuttling mediators between both GOx and GDH-PQQ and the electrodes [50,54–56]. The first research on the amperometric determination of blood glucose using a redox couple-mediated, GOx-catalyzed reaction was demonstrated in 1970 [57]. However, this study did not lead to the rapid application of amperometry in SMBG in the home setting [58].

During the 1980s, mediator-based second-generation glucose biosensors, the introduction of commercial screen-printed strips for SMBG, and the use of modified electrodes and tailored membranes for enhancing the sensor performance were developed and implemented [50,51,59–62]. The first electrochemical blood glucose monitor for self-monitoring of diabetic patients was pen-sized and was launched in 1987 as ExacTech by Medisense Inc. It used GDH-PQQ and a ferrocene derivative [60]. Its success led to a revolution in the health care of diabetic patients. The current operation of most commercial glucose biosensors does not differ significantly from that of the ExacTech meter. Various self-monitoring glucose biosensors are based on the use of ferrocene or ferricyanide mediators.

Various strategies to facilitate electron transfer between the GOx redox center and the electrode surface have been employed, such as enzyme wiring of GOx by electron-conducting redox hydrogels, the chemical modification of GOx with electron-relay groups and the application of nanomaterial as electrical connectors [48,63–65].

### 3.3. Third-generation of Glucose Biosensors

The third-generation glucose biosensors are reagentless and based on direct transfer between the enzyme and the electrode without mediators. Instead of mediators with high toxicity, the electrode can perform direct electron transfers using organic conducting materials based on charge-transfer complexes [66,67]. Therefore, third-generation glucose biosensors have led to implantable, needle-type devices for continuous *in vivo* monitoring of blood glucose. Conducting organic salts, such as tetrathiafulvalene-tetracyanoquinodimethane (TTF-TCNQ), are known to mediate the electrochemistry of pyrrole-quinolinequinone enzymes (GDH-PQQ) as well as of flavoproteins (GOx). And the absence of mediators provides the biosensors with superior selectivity. However, only a few enzymes including peroxidases have been proved to exhibit direct electron transfer at normal electrode surfaces [62,68]. Several studies for other direct electron transfer approaches on the third-generation glucose biosensors have been reported, including TTF-TCNQ that has a tree-like crystal structure [66,67], the GOx/polypyrrole system [66,69,70], and oxidized boron-doped diamond electrodes [71].

## 4. Continuous Glucose Monitoring Systems (CGMS)

Continuous *ex vivo* monitoring of blood glucose was proposed in 1974 [72], while *in vivo* glucose monitoring was demonstrated in 1982 [52]. CGMS would offer an improved control of diabetes in providing real-time data of an internal insulin release system.

Two types of continuous glucose monitoring systems are currently in use - a continuous subcutaneous glucose monitor and a continuous blood glucose monitor. However, due to surface contamination of the electrode by proteins and coagulation factors and the risk of thromboembolism, most of the CGMSs do not measure blood glucose directly. Therefore, subcutaneously implantable needle-type electrodes measuring glucose concentrations in interstitial fluid have been developed, which reflect the blood glucose level [73–77].

Shichiri *et al.* described the first needle-type enzyme electrode for subcutaneous implantation in 1982 [52]. The first commercial needle-type glucose biosensor was marketed by Minimed (Sylmar, CA, USA). However, it did not provide real-time data, the results of 72 hr monitoring could be downloaded in a physician’s office [78]. The FDA-approved, needle-type CGMS devices including Minimed Guardian REAL-Time system by Medtronic (Minneapolis, MN, USA), SEVEN by Dexcom (San Diego, CA, USA) and Freestyle Navigator by Abbott (Abbott Park, IL, USA) are most widely used CGMS on the market. These devices display updated real-time glucose concentrations every one to five minutes. The disposable sensor can be used for three to seven days [79].

Continuous subcutaneous glucose monitoring can also be achieved without direct contact between the interstitial fluid and transducer by using the microdialysis technique [80,81]. GlucoDay (Menarini, Florence, Italy) and SCGM (Roche, Mannheim, Germany) are based on a microdialysis technique. This approach provides both better precision and accuracy, and lower signal drift than needle-type sensors [82,83].

However, numerous requirements of *in vivo* CGMS include biocompatibility, calibration, long-term stability, specificity, linearity, and miniaturization. The accuracy of these innovative devices is lower than that of traditional glucose biosensors. Although CGM can be associated with improved glycemic control in adults and children with type 1 diabetes [84–86], the clinical usefulness of CGMS has not yet been established [87].

## 5. Non-invasive Glucose Monitoring System

Non-invasive glucose analysis is another goal of glucose sensor technology and significant efforts have been made to achieve this goal. Optical or transdermal approaches are the most common noninvasive glucose sensing methods [88,89]. The optical glucose sensors use the physical properties of light in the interstitial fluid or the anterior chamber of the eye. These approaches include polarimetry [90], Raman spectroscopy [91], infrared absorption spectroscopy [92], photo acoustics [93], and optical coherence tomography [94].

The GlucoWatch Biographer, manufactured by Cygnus, Inc. (Redwood City, CA, USA), was the first transdermal glucose sensor approved by the US FDA. This watch-like device was based on transdermal extraction of interstitial fluid by reverse iontophoresis. It never widely accepted in the market due to long warm up time, false alarm, inaccuracy, skin irritation and sweating. It was withdrawn in 2008. Considerable efforts have been made in the development of non-invasive glucose devices. However, reliable non-invasive glucose measuring method is still not available.

## 6. Glucose Biosensors for Pont-of-Care Testing (POCT)

Although laboratory analysis is the most accurate method for evaluating glucose levels, because of cost and time delays, POCT is widely used to determine glucose levels in the inpatient (ER/ICU/ward) and outpatient (office/home) setting. The majority of POC glucose biosensors rely on disposable, screen-printed enzyme electrode test strips [28]. These plastic or paper strips have electrochemical cells and contain GDH-PQQ, GDH-NAD, GDH-FAD, or GOx along with a redox mediator [33]. A test strip is first inserted into the meter, and then a small drop of capillary blood is obtained from the fingertip with a lancing device, and is applied to the test strip. Finally, a conversion factor is applied and the measurement results are typically displayed as plasma glucose equivalents according to the IFCC recommendation [95].

Since the launching of ExacTech in 1987, the portable glucose biosensors have achieved the most significant commercial success. Subsequently, many different devices have been introduced on the global market. The 2010 issue of the Diabetes Forecast Resource Guide, which has a clear focus on the US market, lists 56 different POC glucose sensors from 18 different companies. However, over 90% of the market consists of products manufactured by four major companies, including Abbott, Bayer, LifeScan, and Roche. A brief summary of the key features of commercially available glucose biosensors is provided in Table 2. Most of the meters are plasma-blood calibrated. The measurement requires a 0.3- to 1.5-μL drop of blood and usually takes less than 10 seconds for the result. To choose a glucose biosensor, the practical (ease of use, size of the test strip, amount of blood needed), technical (analytical reliability, testing speed, ability to store test results in memory) and economical (cost of the meter and or the test strips) factors should be considered [96].

Currently, many POC devices can be directly connected to laboratory information systems via proprietary data management system. This significantly expands the data management and networking capabilities of bedside glucose biosensors, and allows for centralized quality control management.

## 7. Analytical Performance Validation of Glucose Biosensors

Glucose biosensors are easy to handle, require minimal amounts of blood, and can perform rapid measurements. Suboptimal measurement quality can lead to significant inaccuracies and increased patient morbidity and mortality. Therefore, a number of criteria for analytical performance have been recommended. In 1994, the ADA made available the first recommendations for the analytical performance of glucose biosensors, suggesting a <10% threshold of maximum allowable bias from reference methods for glucose concentrations between 1.6 and 22.2 mmol/L [97]. This analytical target was further reduced to <5% in 1996 [98]. According to the U.S.FDA recommendations, glucose sensors must have an error of <20% for glucose concentrations between 1.65 and 22 mmol/L when compared to the reference laboratory measurements. The criteria proposed by the International Organization for Standardization (ISO) 15197:2003 are stratified for blood glucose levels <75 or ≥75 mg/dl. Furthermore, 95% of the individual glucose results are required to be within ±0.83 mmol/L (15 mg/dL) of the results of the manufacturer’s measurement procedures at glucose concentrations <4.2 mmol/L (<75 mg/dL) and within ±20% at glucose concentrations ≥4.2 mmol/L (≥75 mg/dL).

For most glucose monitoring devices, the analytical performance has been validated by healthcare professionals according to the Clinical and Laboratory Standards Institute (CLSI) guidelines. However, the ISO Technical Committee ISO/TC 212 released a protocol to validate the accuracy and repeatability of glucose monitoring devices at three to five different glucose levels in the ISO 15197 guideline [99]. These guidelines emphasize the need to evaluate glucose biosensors in real-life situations. In addition, the guidelines specify the requirements for *in vitro* glucose monitoring systems that measure glucose concentrations in capillary blood samples and procedures for the verification and validation of performance by the intended users.

The U.S. FDA guidelines document also provides recommendations for POC glucose biosensors using capillary whole blood. It recommends the study designs, statistical evaluations, and presentations outlined in ISO 15197 for precision, accuracy, and user performance evaluations [99] and in the CLSI for linearity (EP-6A, Evaluation of the Linearity of Quantitative Measurement Procedures: A Statistical Approach; Approved Guideline) [100] and interfering agents (EP-7A, Interference testing in clinical chemistry; Approved guideline) [101]. The guidelines can be summarized as follows in point 7.1–7.5 [99–101].

### 7.1. Precision

#### 7.1.1. Repeatability

Repeatability is evaluated at five glucose concentrations spread across the measuring interval (30–50 mg/dL, 51–110mg/dL, 111–150 mg/dL, 151–250 mg/dL, and 251–400 mg/dL) and should be measured over a short span of time, not to exceed one day per meter, with the same user, meter, and reagent lot. At least 500 reagent system units and 10 meters are required, and the preferred sample for evaluation is venous blood. The samples are equilibrated to a temperature of 23 °C ± 5 °C and maintained within ±2 °C of the starting temperature during the experiments. A total of ten measurements per sample per meter are performed. The mean value, the standard deviation (SD) and the coefficient of variation (CV) for each meter from the ten measurements are calculated. The grand mean, the pooled variance, the pooled SD (with 95 % confidence interval) and the pooled CV are calculated.

#### 7.1.2. Intermediate precision

Intermediate precision is defined as precision under “conditions where the test results are obtained with the same method on identical test items in the same location, but where other variables such as operators, equipment, calibration, environmental conditions, and/or time intervals differ.”

Intermediate precision evaluation is performed with control materials at three glucose concentrations (30–50 mg/dL, 96–144 mg/dL and 280–420 mg/dL). The evaluation is conducted with multiple meters and different users over at least 10 days. At least 300 reagent system units and 10 meters are required. It requires one measurement per day of a sample from each glucose concentration for 10 days for each of 10 meters. The mean, SD and CV for each meter from the ten measurements are calculated. The grand mean, the pooled variance, the pooled SD (with 95 % confidence interval) and the pooled CV are calculated. The SD and CV are measures of the intermediate precision of a single system over multiple days.

### 7.2. Accuracy

System accuracy is evaluated with at least 100 different subjects and two different meters over at least 10 days with capillary blood samples. The glucose concentrations should be distributed as specified in Table 3. Samples are measured by two different meters and at least in duplicate. The evaluation should be conducted in actual conditions of use, so that the effects of systematic error and random error that would be experienced by individual users are included. The results obtained with a glucose biosensor are compared, in a setting representative of intended use, to reference glucose concentration values obtained by a legally marketed device that has been well validated for precision and accuracy. Paired reference laboratory results and results obtained by glucose biosensors are used to calculate the correlation coefficient, regression equation, and the difference in plot analysis.

### 7.3. Linearity

Glucose biosensors should be evaluated for linearity across the reportable range. Therefore, data collection requires two to four replicates from 5 to 11 samples with varying concentrations that are known relative to one another by the dilution ratio or by the formulation. The correlation coefficient and linear regression equations are calculated from the obtained results *versus* glucose concentration values.

### 7.4. User performance

If the glucose biosensors are evaluated by highly trained health care professionals only, they could miss the complications that are likely to occur by patients using the devices [102]. The purpose of user performance evaluation is to demonstrate that users are able to operate the glucose biosensors, given only the instructions and training materials routinely provided with the system and obtain valid glucose results. Results obtained by the lay user are compared to the results obtained by a validated glucose measurement procedure as well as to the results obtained by a healthcare professional from the same sample, using the same devices.

At least 50 subjects, with varying demographics (age, gender, and education level), should be included for each lot. User studies are conducted using at least three different reagent lots at multiple sites. After reviewing the routinely provided training materials, the users perform their own finger sticks and test themselves using the devices. Immediately after the user’s self-test, the investigation site’s trained healthcare professional measures the user’s blood with the devices.

The second blood sample should be collected within five minutes. The results are fitted to regression equations with confidence intervals and plots showing all data points. The results should include a comparison of the user results, professional results, and reference results.

### 7.5. Interferences

A number of variables can influence the reliability of the test results, including hematocrit, hypoxemia, hypotension, altitude, temperature, and humidity. Electrochemical interferents in the blood cause a false high glucose reading by donating non-glucose-derived electrons. An interfering molecule is a species that is electroactive at the operating potential of the amperometric sensor. Suggested standard interferents developed by the FDA include: acetaminophen, salicylic acid, tetracycline, dopamine, ephedrine, ibuprofen, L-DOPA, methy-DOPA, tolazamide, ascorbic acid, bilirubin, cholesterol, creatinine, triglycerides, and uric acid [33].

Hematocrit values have a marked effect on the strip-based glucose assay [103,104]. Oxygen from red blood cells can compete with the redox mediator for glucose-derived electrons in strips when the enzyme used is GOx. Further, the viscosity of blood increases with increasing hematocrit values, and this increase slows the diffusion of all components and reduces the current in the amperometric sensors [105]. Low hematocrit values may be the result of anemia and are associated with overestimated results. Hematocrit causes the most significant error in POC glucose biosensors, especially in the intensive care unit.

Ascorbic acid is one of the most common interfering substances that affect the accuracy of glucose biosensors [106,107]. For glucose biosensors based on electrochemical analysis, ascorbic acid is oxidized at the electrode surface, resulting in the production of more electrons and the generation of a greater current. Increased levels of ascorbic acid lead to increased glucose levels due to the varying degrees of interference caused by ascorbic acid on the glucose biosensors; this may be due to the differences in the enzymes used, technical methodology, or construction of the test strips.

GDH-PQQ catalyzes not only the oxidation of glucose, but also of other sugars, such as maltose, maltriose, maltotetraose, and icodextrin (Extraneal; Baxter, Brussels, Belgium) [108–110]. Most GDH-based POC devices have significant overestimations of glucose in patients undergoing peritoneal dialysis using icodextrin as an osmotic agent [110,111]. Icodextrin is metabolized in the systemic circulation into different glucose polymers, but mainly maltose, which interferes with the GDH-PQQ-based method. The maltose effect on glucose measurement has been demonstrated by Janssen *et al*. [110]. These authors found that icodextrin metabolites can cause positive interference that may lead to a missed diagnosis of hypoglycemia. Clinicians should be aware of this analytical interference. The glucose biosensors based on the GDH-PQQ method should preferably not be used in this high-risk population, and POC glucose results inconsistent with clinical suspicion of hypoglycemic coma should be retested with another testing system.

Various drugs have been shown to interfere with glucose [112]. Acetaminophen is one of the most common drugs associated with both accidental and intentional poisoning. A high dose of acetaminophen can generate analytical interference on electrochemical biosensors [113]. This drug is directly oxidized after diffusing across a porous membrane to the electrode surface, producing an interfering current that increases the glucose reading.

## 8. Conclusions

The measurement of blood glucose levels is carried out using various glucose biosensors for the screening, diagnosis, and long-term management of patients with diabetes. Since the prevalence of diabetes is increasing, novel glucose biosensor technologies, including POC devices, CGMS, and noninvasive glucose monitoring systems, have been developed during the last few decades. Recently, the value of glucose biosensors at the POCT by medical professionals and the SMBG by patients has been widely accepted. Rapid and effective corrections of blood glucose levels are based on regular glucose measurements using glucose biosensors.

Glucose biosensors have evolved to be more reliable, rapid, and accurate and are also more compact and easy to use. Research for advanced technologies, including electrodes, membrane, immobilization strategies, and nanomaterials, continue to be performed. Despite the impressive advances in glucose biosensor technology, there are still several challenges related to the achievement of reliable glucose monitoring. The ADA recommends the accuracy of a blood glucose POC assay to be <5% of the measured value. However, many POC devices do not meet this criterion. Biosensor technology is less precise and less accurate than the methods used in central laboratories [114].

A more systematic evaluation of the analytical performance of glucose biosensors is recommended to ensure reliable and accurate testing. Analytical requirements for suitable hospital or home POC devices include good linearity, precision, and correlation when compared to a clinical laboratory reference method as well as resistance to common interferences. The calibration of the devices and quality control should be performed on a regular basis according to the manufacturer’s instructions. User-dependent factors can also affect data quality, and by extension, treatment outcomes. The most commonly cited problems are incorrect use of the test strip, lack of quality control procedure, fingers that are not clean and dirty devices. Various studies have shown that education and continuous training can reduce errors caused by the aforementioned factors and improve measurement performance [115].

Therefore, in addition to further technical improvements of the biosensors, standardization of the analytical goals for improved performance, and continuous assessment and training of lay users should be established.

## Figures and Tables

**Table 1.** History of glucose biosensors.

Year	Event
1962	First description of a biosensor by Clark and Lyons
1967	First practical enzyme electrode by Updike and Hicks
1973	Glucose enzyme electrode based on detection of hydrogen peroxide [36]
1975	Relaunch of first commercial biosensor, *i.e.*, YSI analyzer
1976	First bedside artificial pancreas (Miles)
1982	First needle-type enzyme electrode for subcutaneous implantation by Shichiri
1984	First ferrocene mediated amperometric glucose biosensor by Cass
1987	Launch of the MediSense ExacTech blood glucose biosensor
1999	Launch of a commercial *in vivo* glucose sensor (MiniMed)
2000	Introduction of a wearable noninvasive glucose monitor (GlucoWatch)

**Table 2.** Commercially available glucose biosensors.

Manufacturer	Brand	Assay method	Minimal sample volume (uL)	Test time (second)	Assay range (mg/dL)	Hematocrit range (%)	Memory (results)
Abbott	FreeStyle Freedom Lite	GDH-PQQ	0.3	−5	20–500	15–65	400
AgaMatrix	WaveSense KeyNote	GOD	0.5	4	20–600	20–60	300
Arkray	Glucocard X-meter	GDH	0.3	5	10–600	30–52	360
Bayer	Ascensia Contour	GDH-FAD	0.6	5	10–600	0–70	480
Bionime	Rightest GM300	GOD	1.4	8	20–600	30–55	300
Diabestic Supply of Suncoast	Advocate Redi-Code*	GOD	0.7	7	20–600	20–60	450
Diagnostic Devices	Prodigy Autocode	GOD	0.6	6	20–600	20–60	450
LifeScan	OneTouch UltraLink	GOD	1.0	5	20–600	30–55	500
Nova Biomedical	Nova Max	GOD	0.3	5	20–600	25–60	400
Roche	Accu-Chek Aviva	GDH-PQQ	0.6	5	10–600	20–70	500

* This monitor has audio features to help visually impaired use.

**Table 3.** Glucose concentrations of samples for the evaluation of system accuracy [99].

Percentage of samples (%)	Glucose concentration (mg/dL)
5	<50
15	50–80
20	80–120
30	120–200
15	201–300
10	301–400
5	>400
