# Correction: Yoo, E.H., *et al*. Glucose Biosensors: An Overview of Use in Clinical Practice. *Sensors* 2010, *10*, 4558–4576

**Published:** 2010-08-23

**Authors:** Eun-Hyung Yoo, Soo-Youn Lee

**Affiliations:** 1 Department of Laboratory Medicine, Konyang University Hospital, College of Medical Science, Konyang University, Daejon, Korea; E-Mail: yooeh@kyuh.co.kr; 2 Department of Laboratory Medicine and Genetics, Samsung Medical Center, Sungkyunkwan University School of Medicine, Seoul, Korea

The authors would like to correct the Table 2 of this paper [1] as follows:

**Table 2.** Commercially available glucose biosensors

Manufacturer	Brand	Assay method	Minimal sample volume (uL)	Test time (second)	Assay range (mg/dL)	Hematocrit range (%)	Memory (results)
Abbott	FreeStyle Freedom Lite	GDH-PQQ	0.3	∼5	20∼500	15∼65	400
AgaMatrix	WaveSense KeyNote	GOD	0.5	4	20∼600	20∼60	300
Arkray	Glucocard X-meter	GDH	0.3	5	10∼600	30∼52	360
Bayer	Ascensia Contour	GDH-FAD	0.6	5	10∼600	0∼70	480
Bionime	Rightest GM300	GOD	1.4	8	20∼600	30∼55	300
Diabestic Supply of Suncoast	Advocate Redi-Code*	GOD	0.7	7	20∼600	20∼60	450
Diagnostic Devices	Prodigy Autocode*	GOD	0.6	6	20∼600	20∼60	450
LifeScan	OneTouch UltraLink	GOD	1.0	5	20∼600	30∼55	500
Nova Biomedical	Nova Max	GOD	0.3	5	20∼600	25∼60	400
Roche	Accu-Chek Aviva	GDH-PQQ	0.6	5	10∼600	20∼70	500

* These monitors have audio features to help visually impaired use
